# Evaluation of root canal morphology of premolar teeth using cone beam computed tomography: a retrospective study

**DOI:** 10.1186/s12903-026-07929-z

**Published:** 2026-02-24

**Authors:** Tuğba Cebeci, Berceste Polat Akmansoy

**Affiliations:** Department of Oral and Maxillofacial Radiology, Marmara University Recep Tayyip Erdoğan Complex, Health Campus, Faculty of Dentistry, Başıbüyük/Maltepe/Istanbul, 9/3 34854 Turkey

**Keywords:** Cone-Beam computed tomography, Premolar teeth, Root canal anatomy

## Abstract

**Background:**

This study aimed to evaluate the root and canal morphology of maxillary and mandibular premolars using cone-beam computed tomography (CBCT) and to assess bilateral anatomical symmetry between contralateral teeth.

**Methods:**

A total of 157 CBCT scans from patients aged 18–60 years were retrospectively analyzed. Root number, canal number, Vertucci classification types, and bilateral symmetry of premolars in the maxilla and mandible were recorded.

**Results:**

Maxillary first premolars most commonly exhibited two roots and two canals (70.70%), with Vertucci Type IV being the dominant configuration (78.02%). Maxillary second premolars most frequently presented one root with two canals (48.40%), and Vertucci Type I was the most prevalent morphology (28.66%). Mandibular first and second premolars predominantly exhibited one root and one canal (72.29% and 93.63%), with Vertucci Type I being the most frequent configuration (71.65% and 93.63%). Bilateral root and canal symmetry was high for all premolars: 89.15% in maxillary first premolars, 89.78% in maxillary second premolars, 87.26% in mandibular first premolars, and 94.90% in mandibular second premolars. Root and canal morphology differed by sex and by jaw (*p* < 0.001).

**Conclusions:**

CBCT enables accurate visualization of premolar root canal anatomy and assists clinicians in identifying anatomical variations. The high degree of bilateral symmetry suggests that contralateral teeth may serve as useful anatomical references during endodontic procedures.

## Introduction

Endodontic treatment aims to preserve tooth structure by addressing diseases affecting the dental pulp and periapical tissues [1]. The success of root canal treatment depends on effective eradication of microorganisms through chemical irrigation and mechanical debridement [2]. A detailed understanding of root canal morphology, including anatomical variations, is essential for improving treatment outcomes [3–6].

**Fig. 1:**
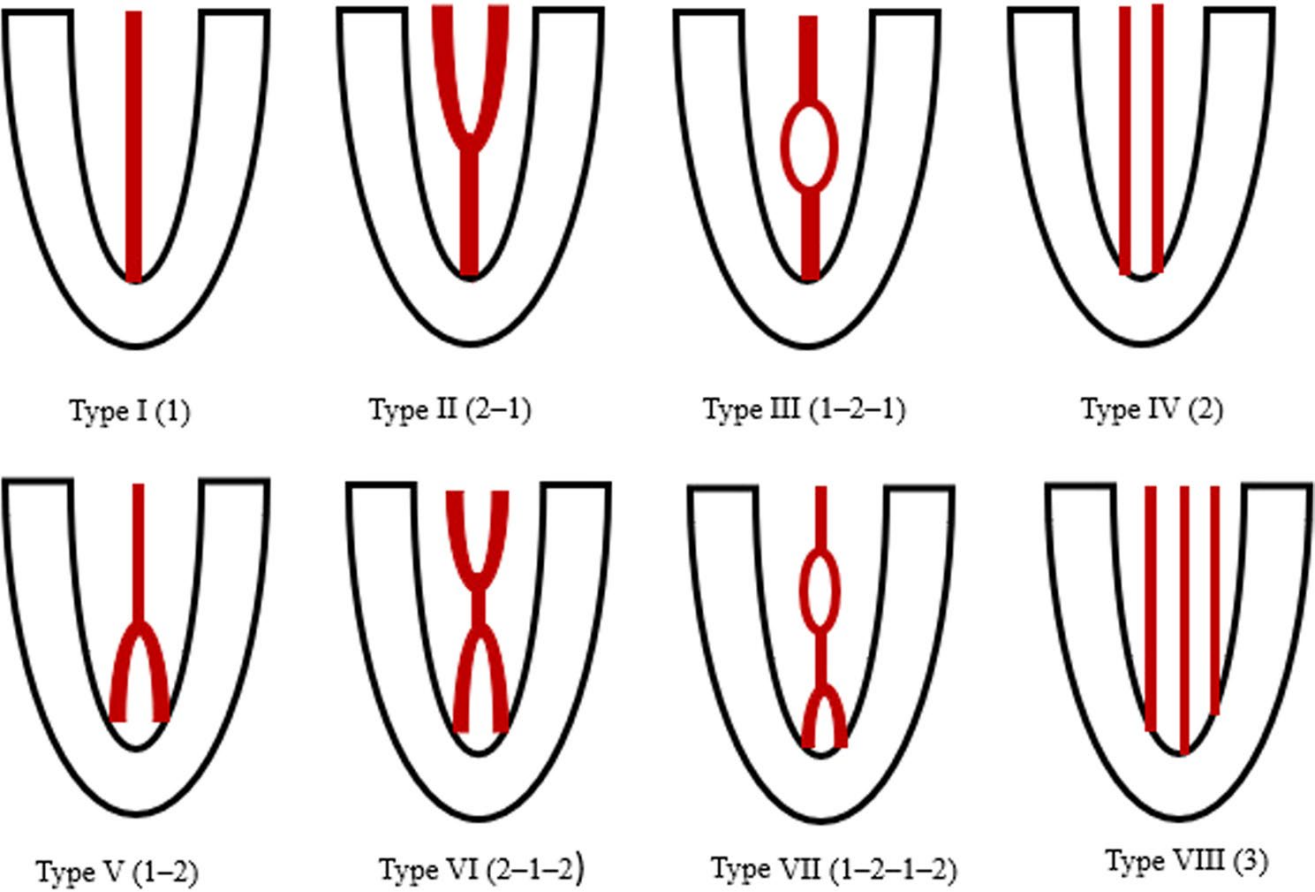
Schematic illustration of Vertucci’s root canal configuration types (I–VIII)

Variations in the internal anatomy of root canals may result in untreated areas, increasing the likelihood of endodontic complications and treatment failure [7–9]. Root canal systems show considerable complexity, with bifurcations, divisions, and subsequent reconvergence occurring frequently [10]. Vertucci’s classification system provides a widely accepted framework for describing root canal morphology by categorizing canal configurations into eight distinct types based on their internal anatomy. In Type I, a single root canal extends continuously from the pulp chamber to the apical foramen. Type II is characterized by two distinct canals that originate from the pulp chamber and merge into a single canal before reaching the apex. In Type III, one canal leaves the pulp chamber, divides within the root, and subsequently reunites to exit as a single canal at the apex. Type IV consists of two separate and independent canals extending from the pulp chamber to the apex without merging. In Type V, a single canal divides near the apex into two separate canals, each with its own apical foramen. Type VI involves two canals that arise from the pulp chamber, merge within the root, and then divide again before exiting as two distinct canals at the apex. In Type VII, one canal originates from the pulp chamber, divides and rejoins within the root, and finally separates again into two distinct canals prior to the apex. Type VIII is defined by the presence of three separate and independent canals extending from the pulp chamber to the apex [11] (Fig. 1).

Premolars often present with complex canal anatomies, multiple roots, and varied configurations, making them particularly challenging during endodontic procedures [12–14]. Although periapical radiographs are commonly used for evaluating root morphology, their two-dimensional nature introduces limitations, including superimposition and distortion. As two-dimensional imaging cannot always accurately reveal the true number of canals, undetected anatomical structures may result in incomplete disinfection of the canal system and subsequently poorer treatment outcomes [15–20]. Cone-beam computed tomography (CBCT) offers a noninvasive alternative that enables high-resolution, three-dimensional evaluation of maxillofacial structures in axial, sagittal, and coronal planes, eliminating the issue of superimposition inherent to conventional radiography [19, 21–23].

Preoperative identification of the number and spatial orientation of root canals assists clinicians in accurately locating canal orifices and optimizing access cavity design. Precise characterization of root canal anatomy enhances the efficiency of canal negotiation, cleaning, and obturation, ultimately improving the prognosis of endodontic treatment [24].

The purpose of this study was to evaluate the root anatomy and canal morphology of maxillary and mandibular first and second premolars in a Turkish population using CBCT.

## Materials and methods

### Ethical considerations

This retrospective study was conducted in accordance with the ethical principles outlined in the 2008 Declaration of Helsinki and was approved by the Clinical Research Ethics Committee of Marmara University, Faculty of Medicine (Project No: 09.2023.709).

## Study design and participants

The study population consisted of patients who underwent CBCT imaging at the Department of Oral and Maxillofacial Radiology, Faculty of Dentistry, Marmara University between January 2021 and April 2023. The CBCT scans had been obtained for various diagnostic indications, including the assessment of dentoalveolar trauma, evaluation of impacted teeth, and preoperative implant planning.

Maxillary and mandibular premolars were included according to the following criteria:

1. Fully developed permanent premolars with closed apices
2. Absence of root resorption, pulp canal calcifications, or periapical pathology
3. No history of endodontic treatment, post placement, or full-coverage crown restoration
4. Availability of high-resolution CBCT images of diagnostic quality
5. Presence of all premolars within the dental arch
6. Complete visualization of all premolars within the CBCT field of view (FOV)

Individuals of non-Turkish ethnic origin were excluded.

### Radiographic evaluation and data collection

CBCT imaging was performed using a Planmeca^®^ ProMax 3D Mid unit (Planmeca Oy, Helsinki, Finland) with a 16 × 9 cm field of view, an isotropic voxel size of 0.4 mm³, and a slice thickness of 0.40 mm. Scans were acquired at 90 kVp and 10 mA over an exposure time of 36 s. All images were exported in Digital Imaging and Communications in Medicine (DICOM) format and evaluated using Romexis^®^ imaging software (Planmeca Oy, Helsinki, Finland).

A total of 6,519 CBCT examinations were screened. Of these, 165 CBCT scans met the inclusion criteria. Two experienced oral and maxillofacial radiologists independently evaluated the CBCT scans in axial, coronal, sagittal, and cross-sectional planes using multiplanar reconstruction. Consecutive slices in each plane were reviewed to assess root number and root canal morphology along the entire root length. In instances of inter-observer disagreement, the images were reviewed jointly through discussion until a consensus was reached. When consensus could not be achieved for any premolar tooth, the corresponding CBCT examination was excluded, as all premolar teeth within each individual were evaluated collectively. Therefore, the exclusion of 8 premolar teeth resulted in the exclusion of 8 CBCT scans. The final study sample consisted of 157 individuals aged 18–60 years, from whom a total of 1,256 premolar teeth were included in the analysis.

**Fig. 2:**
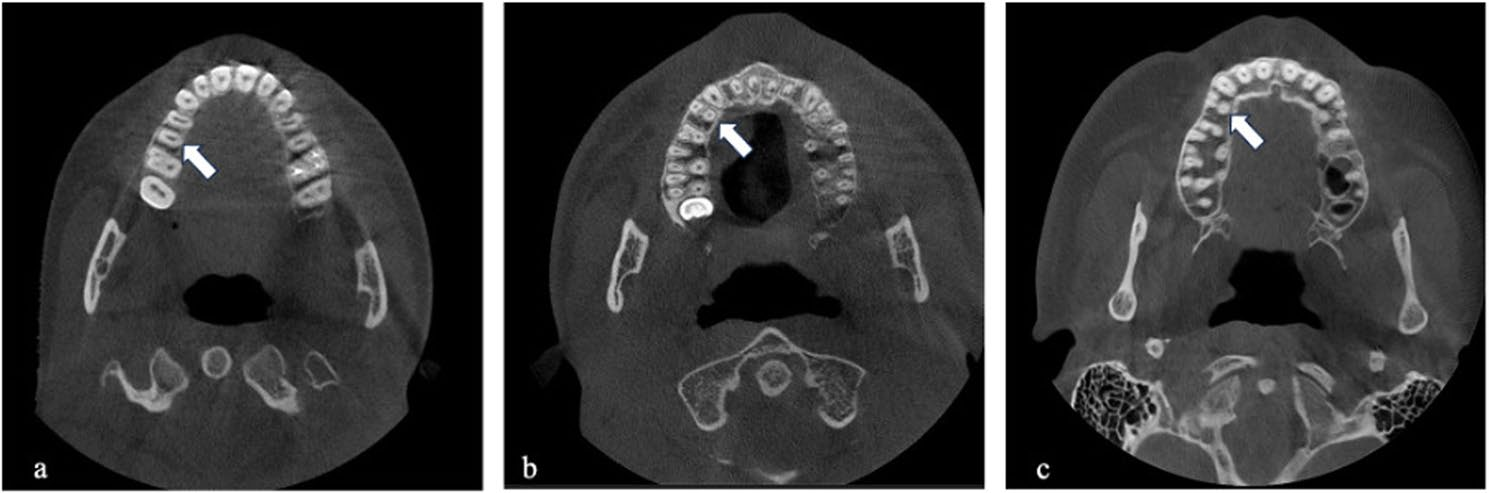
Axial CBCT images of the number of roots only in maxillary premolar teeth. (**a**) One root, (**b**) two roots, (**c**) three roots

**Fig. 3:**
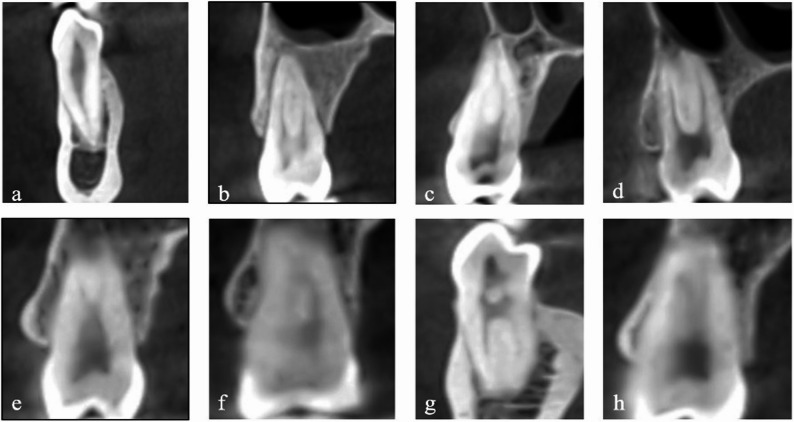
Cross-sectional CBCT images illustrating Vertucci’s root canal configurations: (**a**) Type I (1), (**b**) Type II (2–1), (**c**) Type III (1–2–1), (**d**) Type IV (2), (**e**) Type V (1–2), (**f**) Type VI (2–1–2), (**g**) Type VII (1–2–1–2), and (**h**) Type VIII (3)

All CBCT images were evaluated under standardized viewing conditions using a 22-inch Dell monitor (resolution: 1920 × 1080 pixels; Dell Inc., Round Rock, TX, USA) in a dimly lit room at a viewing distance of approximately 40–50 cm. Prior to image evaluation, the observers reached a consensus regarding contrast, brightness, and magnification settings to optimize visualization. Following this initial calibration, the agreed-upon display settings were applied consistently across all CBCT scans. These adjustments were limited to display parameters only and did not involve any modification of the original image data. The following parameters were recorded for each tooth:

1. Number of roots (Fig. 2) and root canals. The number of root canals was defined as the total number of distinct canals extending from the pulp chamber to the apical foramen, independent of the number of roots.
2. Root canal configurations were classified according to Vertucci’s classification system. The canal pathway patterns for each Vertucci type were defined as follows: Type I (1), Type II (2–1), Type III (1–2–1), Type IV (2), Type V (1–2), Type VI (2–1–2), Type VII (1–2–1–2), and Type VIII (3) (Fig. 3).
3. Bilateral anatomical symmetry. It was defined as the presence of the same Vertucci canal configuration in contralateral premolar teeth within the same individual. The symmetry rate for each Vertucci classification type was calculated as the proportion of bilaterally symmetrical cases of that specific configuration relative to the total number of teeth exhibiting bilateral symmetry.

### Statistical analysis

IBM SPSS Statistics for Windows, version 20.0 (IBM Corp., Armonk, NY, USA) was used for data analysis. Descriptive statistics were presented as frequencies and percentages. Associations between categorical variables were assessed using the Chi-square test. Statistical significance was set at *p* < 0.05. A post hoc power analysis was performed using G*Power (α = 0.05) and indicated adequate power for the primary categorical comparisons (achieved power > 0.80). Intra- and interobserver reliability were evaluated using Cohen’s kappa coefficient on a randomly selected subset of 20 CBCT scans; interobserver agreement was based on the observers’ independent assessments, and intraobserver agreement was determined by repeat evaluation after a 2-week interval (κ = 0.86 and κ = 0.91, respectively).

## Results

Vertucci Type IV was the most frequently observed canal configuration in maxillary first premolars (Table 1). Most of these teeth presented with two roots (Table 1), and the presence of two canals was the most common finding (Table 1). Evaluation of combined root and canal morphology showed that 4.14% of teeth exhibited one root–one canal, 24.20% had one root–two canals, 70.70% had two roots–two canals, and 0.95% had three roots–three canals. Bilateral symmetry according to Vertucci’s classification was identified in 91.71% of cases. Symmetry rates for each configuration type were as follows: Type I (3.18%), Type II (7.64%), Type III (5.09%), Type IV (74.52%), Type V (0.63%), and Type VIII (0.63%), while Types VI and VII showed no symmetry. Overall, root and canal morphology demonstrated bilateral symmetry in 89.15% of patients, with symmetrical distributions of one root–one canal (3.18%), one root–two canals (19.10%), two roots–two canals (66.24%), and three roots–three canals (0.63%).

**Table 1:** Distribution of root number, canal number, and Vertucci canal configurations by FDI Notation Teeth Number

	Number of Roots(%)			Number of Root Canals (%)			Vertucci Classification (%)							
	One	Two	Three	One	Two	Three	Type I	Type II	Type III	Type IV	Type V	Type VI	Type VII	TypeVIII
Maxillary 1st Premolar	26.43%	72.61%	0.95%	3.82%	95.22%	0.95%	3.82%	8.91%	6.68%	78.02%	0.95%	0.63%	0%	0.63%
Maxillary 2nd Premolar	78.34%	21.01%	0.63%	28.66%	70.70%	0.63%	28.66%	14.33%	24.84%	26.43%	4.45%	0.63%	0%	0.63%
Mandibular 1st Premolar	76.43%	23.56%	0%	71.65%	28.35%	0%	71.65%	0%	1.59%	0%	26.43%	0%	0.31%	0%
Mandibular 2nd Premolar	96.17%	3.82%	0%	93.63%	6.37%	0%	93.63%	0%	1.59%	0%	4.77%	0%	0%	0%

The predominant root canal configuration in maxillary second premolars was Vertucci Type I (Table 1). One root was most commonly observed (78.34%) (Table 1), and two canals represented the most frequent canal number (70.70%) (Table 1). Detailed assessment showed that 28.34% of teeth had one root–one canal, 48.40% had one root–two canals, 0.31% had one root–three canals, 22.29% had two roots–two canals, and 0.63% had three roots–three canals. Bilateral symmetry according to Vertucci’s classification was observed in 91.71% of cases. The distribution of symmetrical types was: Type I (28.66%), Type II (14.33%), Type III (24.84%), Type IV (26.43%), Type V (4.5%), Type VI (0.63%), Type VII (0%), and Type VIII (0.63%). Bilateral root and canal symmetry was found in 89.78% of teeth, including one root–one canal (25.47%), one root–two canals (44.58%), two roots–two canals (19.10%), and three roots–three canals (0.63%).

The most prevalent canal configuration in mandibular first premolars was Vertucci Type I (Table 1). A single root was the most frequently observed morphology (Table 1), and one canal was the most common finding (Table 1). Detailed analysis revealed that 72.29% of teeth had one root–one canal, 5.73% had one root–two canals, and 21.97% had two roots–two canals. Bilateral Vertucci symmetry was present in 86.62% of cases. Symmetry rates by configuration type were: Type I (24.84%), Type II (11.46%), Type III (21.65%), Type IV (24.20%), Type V (3.18%), Type VI (0%), Type VII (0%), and Type VIII (0.63%). Root and canal morphology demonstrated bilateral symmetry in 87.26% of patients, including one root–one canal (66.24%), one root–two canals (3.18%), and two roots–two canals (17.83%).

Vertucci Type I was the predominant canal configuration in mandibular second premolars (Table 1). One root was most frequently observed (Table 1), and one canal represented the most common canal morphology (Table 1). Detailed evaluation showed that 93.63% of teeth exhibited one root–one canal, 2.22% had one root–two canals, and 4.14% had two roots–two canals. Bilateral symmetry based on Vertucci’s classification was identified in 94.90% of patients. Symmetry rates by configuration type included: Type I (65.60%), Type III (1.27%), and Type V (19.74%), while Types II, IV, VI, VII, and VIII were not observed. Bilateral root and canal morphology symmetry was also present in 94.90% of teeth, with symmetrical patterns including one root–one canal (91.08%), one root–two canals (1.27%), and two roots–two canals (2.5%).

Sex and jaw-based comparisons revealed significant differences in premolar root and canal morphology (Tables 2 and 3). When analyzed by sex, single-rooted teeth were more frequent in women than in men, whereas the prevalence of two-rooted teeth was higher in men. Women more commonly exhibited a single canal, while men showed a higher prevalence of two canals. With respect to Vertucci canal configurations, Type I was more prevalent in women, whereas Types IV and V were observed more frequently in men, and these differences were statistically significant (*p* < 0.001 for all comparisons).

**Table 2:** Distribution of root number, Canal number, and Vertucci Canal configurations by sex

			Men (*n* = 464)		Women (*n* = 792)		Total (*n* = 1256)		*p*
Root Number		One root	281 (60.6%)		592 (74.7%)		873 (69.5%)		
		Two roots	178 (38.4%)		200 (25.3%)		378 (30%)		*p* < 0.001
		Three roots	5 (1.1%)		1 (0.1%)		6 (0.5%)		
Root Canal Number		One canal	183 (39.4%)		437 (55.2%)		620 (49.4%)		
		Two canals	276 (59.5%)		354 (44.7%)		630 (50.2%)		*p* < 0.001
		Three canals	5 (1.1%)		1 (0.1%)		6 (0.5%)		
Vertucci Classification		Type I	183 (39.4%)		437 (55.2%)		620 (49.4%)		
		Type II	31 (6.7%)		42 (5.3%)		73 (5.8%)		
		Type III	35 (7.5%)		74 (9.3%)		109 (8.7%)		
		Type IV	140 (30.2%)		188 (23.7%)		328 (26.1%)		*p* < 0.001
		Type V	67 (14.4%)		48 (6.1%)		115 (9.2%)		
		Type VI	2 (0.4%)		2 (0.3%)		4 (0.3%)		
		Type VII	1 (0.2%)		0 (0%)		1 (0.1%)		
		Type VIII	5 (1.1%)		1 (0.1%)		6 (0.5%)		

**Table 3:** Distribution of root number, Canal number, and Vertucci Canal configurations by jaw

					Maxilla (*n* = 628)		Mandible (*n* = 628)	Total (*n* = 1256)		*p*
Root Number				One root	329 (52.4%)		544 (86.6%)	873 (69.5%)		
				Two roots	294 (46.8%)		84 (13.4%)	378 (30%)		*p* < 0.001
				Three roots	5 (0.8%)		0 (0%)	5 (0.5%)		
Root Canal Number				One canal	103 (16.4%)		517 (82.3%)	620 (49.4%)		
				Two canals	519 (82.7%)		111 (17.7%)	630 (50.2%)		*p* < 0.001
				Three canals	6 (1%)		0 (0%)	6 (0.5%)		
Vertucci Classification				Type I	103 (16.4%)		517 (82.3%)	620 (49.4%)		
				Type II	73 (11.6%)		0 (0%)	73 (5.8%)		
				Type III	98 (15.6%)		11 (1.8%)	109 (8.7%)		
				Type IV	327 (52.1%)		1 (0.2%)	328 (26.1%)		*p* < 0.001
				Type V	17 (2.7%)		98 (15.6%)	115 (9.2%)		
				Type VI	4 (0.6%)		0 (0%)	4 (0.3%)		
				Type VII	0 (0%)		1 (0.2%)	1 (0.1%)		
				Type VIII	6 (1%)		0 (0%)	6 (0.5%)		

Jaw-based analysis demonstrated marked anatomical differences between the maxilla and mandible. In the maxilla, two-rooted teeth and two canals predominated, and Vertucci Type IV was the most common canal configuration. In contrast, mandibular premolars were predominantly single-rooted with a single canal, and Vertucci Type I was overwhelmingly prevalent. Differences in root number, canal number, and Vertucci canal configurations between the maxilla and mandible were statistically significant (*p* < 0.001 for all comparisons).

## Discussion

Conventional imaging techniques compress three-dimensional anatomical structures into two-dimensional images or shadowgraphs, which significantly limits diagnostic performance. Consequently, the spatial relationship between tooth roots, surrounding anatomical structures, and associated periradicular lesions cannot always be accurately assessed using conventional intraoral radiographs alone. For this reason, alternative imaging techniques have been proposed to overcome the inherent limitations of two-dimensional radiography [25].

Micro-computed tomography (micro-CT) has been widely used in dental research to characterize root and canal configurations, radicular grooves, accessory canals, and apical foramina, providing detailed three-dimensional visualization that enhances the understanding of complex root canal morphology. However, scanning and reconstruction processes in micro-CT technology require relatively long acquisition times, advanced computer expertise, and high operational costs. In addition, the high radiation dose associated with micro-CT prevents its use in clinical settings, and its application is restricted to in vitro studies due to radiation exposure and specimen size limitations. In this context, despite its lower spatial resolution compared with micro-CT, CBCT may represent a more clinically applicable imaging modality. CBCT addresses several limitations of conventional radiography by providing three-dimensional visualization of teeth and surrounding structures, thereby facilitating improved assessment of root canal anatomy in multiple planes without anatomical superimposition [26–29].

Comparisons of studies conducted within the same ethnic population using different imaging modalities further highlight the influence of imaging technique on reported root canal morphology. In a Saudi Arabian population evaluated using periapical radiographs, Al-Nazhan et al. [30] reported that 39.7% of premolars had one canal, 59.4% had two canals, and 0.9% had three canals. In contrast, a CBCT-based study in the same population by Elkady and Allouba [31] demonstrated a higher prevalence of two canals (63.7%) and a lower prevalence of single canals (36.3%). Moreover, micro-CT evaluation of premolars in a Saudi Arabian population revealed an even greater detection of canal complexity, with 65% of teeth presenting two canals, followed by 30% with one canal and 5% with three canals [32]. These findings suggest that differences in reported canal configurations within the same ethnic group may be influenced not only by population characteristics but also by the spatial resolution and diagnostic capabilities of the imaging modality employed, with higher-resolution techniques potentially allowing more accurate identification of complex canal anatomy.

The morphology of maxillary first premolars demonstrated considerable variability in the present study, with a predominance of two-rooted teeth and two canals. While single-rooted maxillary first premolars have been reported at rates of 69.7% in a Chinese population [1] and 51% in a Turkish population [23], higher frequencies of two-rooted teeth have been observed in Brazilian and Saudi Arabian populations, with reported rates ranging from 57.5% to 80.2% [16, 33, 34]. Consistent with these latter findings, two roots were most frequently observed in the present cohort. In terms of canal number, two canals represented the most common configuration, in agreement with previous CBCT-based studies [1, 23, 33, 34]. Vertucci Type IV was identified as the predominant canal configuration, consistent with earlier reports, although prevalence rates varied substantially across studies (42.7%–82.2%). Bilateral symmetry rates for Vertucci configurations were higher in the present study (91.71%) compared with those reported by Li et al. [1] (72.1%), Tian et al. in a Chinese population [22] (64%), and Aljawhar et al. in an Iraqi population [35] (56.6%). Similarly, the overall root canal symmetry rate observed in the present study (89.15%) exceeded those reported by Li et al. [1] (80.2%) and Felsypremila et al. in an Indian population [36] (81.5%).

Maxillary second premolars in the present study predominantly exhibited a single root, a finding that aligns with the consistently high prevalence of single-rooted morphology reported across diverse populations [1, 16, 23, 33, 34]. Although two canals were observed more frequently in the present cohort compared with some previous reports [1, 23, 33], similar frequencies were reported by Mashyakhy [34]. While Vertucci Type I configuration has been commonly reported as the most prevalent pattern in earlier studies [1, 16, 23, 25, 26], its prevalence was notably lower in the present study. Bilateral symmetry based on Vertucci classification was high (91.71%) and exceeded rates reported in Chinese and Iraqi populations [1, 35]. Likewise, overall root canal symmetry (89.78%) was higher than that reported in previous CBCT-based studies [1, 36]. Similarly, overall root canal symmetry in the present study (89.78%) exceeded the symmetry rates reported by Li et al. [1] (81.8%) and Felsypremila et al. [36] (81.5%).

Mandibular first premolars have generally been described as predominantly single-rooted teeth in the literature. High prevalences of single-rooted morphology have been reported in Turkish, Saudi Arabian, Thai, and Iranian populations, with rates ranging from 88.69% to 98.1% [23, 33, 37, 38]. In contrast, the prevalence observed in the present study was comparatively lower, suggesting the presence of population-related anatomical variation. A similar pattern was observed for canal number, as single canals were less frequent in our sample than the higher rates reported in previous studies [9, 23, 38]. Despite these differences, Vertucci Type I remained the predominant canal configuration across studies, although reported frequencies varied widely, ranging from 63% to 92.8% [9, 23, 25, 37, 38]. The prevalence of Type I in the present study was toward the lower end of this range. Bilateral root canal symmetry observed in our study (87.26%) was slightly lower than that reported by Felsypremila et al. [36] (96.1%), yet remained high overall.

Mandibular second premolars exhibited a more consistent morphological pattern across populations. Most studies have reported a very high prevalence of single-rooted morphology, with rates exceeding 88% and often approaching 100% [23, 33, 37–39]. The findings of the present study were in agreement with these reports. Likewise, the prevalence of single canals closely aligned with those reported in previous investigations [23, 38]. Vertucci Type I canal configuration was overwhelmingly dominant in mandibular second premolars in the literature, with reported prevalence rates ranging from 83.6% to 98% [23, 33, 37–39], and comparably high frequencies were also observed in the present study. Bilateral symmetry observed in our sample (94.90%) was comparable to the rates reported by Felsypremila et al. [36] and Alghamdi et al. [39]. Across studies, the most common symmetrical morphology was consistently characterized by one root with one canal.

The relationship between sex and premolar root and canal morphology remains controversial in the literature. Several investigations have reported no statistically significant association between sex and overall canal configuration or root number. Olczak et al. [6], for example, found no significant relationship between sex and the general distribution of Vertucci canal types (*p* > 0.05), although certain configurations exhibited sex-related predominance, with Types I, II, III, and VII occurring more frequently in females and Types IV, V, and VI in males. Similarly, Wu et al. [7] reported no significant differences between males and females in terms of root number or root canal morphology in maxillary and mandibular first premolars (*p* > 0.05). Alenezi et al. [13] also observed no significant sex-related differences in root number for mandibular first and second premolars (*p* > 0.05). In contrast, other studies have demonstrated significant associations between sex and specific morphological parameters. Erkan et al. [23] reported a significant sex-related difference in Vertucci canal configurations, with Type I being more prevalent in females, although no significant difference was noted in canal number. Likewise, Al-Zubaidi et al. [33] identified statistically significant differences between sexes in both the number of roots and the number of root canals in maxillary premolars (*p* < 0.05). In the present study, statistically significant associations were observed between sex and root number, canal number, and Vertucci canal configurations (*p* < 0.001 for all comparisons), indicating that sex may play a meaningful role in premolar root and canal morphology within the studied population. The discrepancies among published findings may be attributed to differences in population characteristics, sample size, tooth grouping strategies, and imaging modalities used for morphological assessment.

The variations observed among studies conducted in different populations suggest that root and root canal morphology may exhibit population-specific characteristics rather than a uniform distribution across all groups. Differences reported between studies from Chinese, Turkish, Brazilian, Saudi Arabian, Iranian, Thai, and Indian populations indicate that ethnic background, genetic predisposition, and developmental factors may partially influence root number, canal number, and canal configuration. In addition, environmental factors, sample characteristics, and methodological differences—particularly imaging modality and evaluation criteria—may further contribute to the variability reported across studies. Therefore, the differences observed between the present findings and previous reports should be interpreted cautiously and underscore the relevance of population-based anatomical data for informed endodontic diagnosis and treatment planning.

### Study limitations

Although CBCT provides reliable three-dimensional visualization of root and canal morphology, the use of a voxel size of 0.4 mm may have limited spatial resolution. Consequently, fine anatomical details such as accessory canals, small canal bifurcations, or subtle canal irregularities may not have been fully visualized. In addition, the present study was conducted at a single center and included individuals from a single ethnic background, which may limit the generalizability of the findings to other populations. Because this was a retrospective CBCT-based study, access to comprehensive medical histories was limited. Therefore, the potential influence of patients’ systemic diseases on root and canal morphology could not be evaluated.

### Clinical implications

The observed variations in premolar anatomy highlight the necessity for clinicians to perform meticulous preoperative assessments. The relatively high prevalence of complex configurations, especially in maxillary premolars, reinforces the importance of considering additional canals during endodontic procedures. The consistently high bilateral symmetry across all premolar groups suggests that the contralateral tooth may serve as a valuable anatomical reference when root canal morphology is unclear. Moreover, the findings support the selective use of CBCT to improve diagnostic accuracy in cases with suspected anatomical complexity. Awareness of these variations can help reduce the likelihood of missed canals and improve endodontic treatment outcomes. In addition to its clinical relevance, the present findings may also contribute to undergraduate and postgraduate dental education by increasing awareness of premolar root canal complexity and bilateral symmetry, thereby supporting more accurate diagnosis and treatment planning during endodontic training.

## Conclusion

This study provides comprehensive insight into the root and canal morphology of maxillary and mandibular premolars in a specific population using CBCT. The findings confirm previously reported anatomical patterns while also identifying notable population-specific variations. High bilateral symmetry across premolar groups enhances clinical predictability, offering valuable guidance when assessing contralateral teeth. Given the observed morphological complexity—particularly in maxillary premolars—careful radiographic evaluation, including selective CBCT use, is essential for accurate diagnosis and successful endodontic treatment. Future multicenter and multiethnic studies are recommended to further validate and expand these findings.

## Data Availability

The datasets used and/or analysed during the current study are available from the corresponding author upon reasonable request.
